# Efficacy, Safety, and Quality of Treatment Satisfaction of Premixed Human and Analogue Insulin Regimens in a Large Cohort of Type 2 Diabetic Patients: PROGENS BENEFIT Observational Study

**DOI:** 10.1155/2018/6536178

**Published:** 2018-03-05

**Authors:** Katarzyna Nabrdalik, Hanna Kwiendacz, Tomasz Sawczyn, Andrzej Tomasik, Michał Kukla, Małgorzata Masierek, Janusz Gumprecht

**Affiliations:** ^1^Department of Internal Medicine, Diabetology and Nephrology in Zabrze, School of Medicine with the Division of Dentistry in Zabrze, Medical University of Silesia, Katowice, Poland; ^2^Department of Physiology in Zabrze, School of Medicine with the Division of Dentistry in Zabrze, Medical University of Silesia, Katowice, Poland; ^3^Second Department of Cardiology, School of Medicine with the Division of Dentistry in Zabrze, Medical University of Silesia, Katowice, Poland; ^4^Department of Gastroenterology and Hepatology in Katowice, Medical University of Silesia, Katowice, Poland; ^5^BIOTON S.A., Warsaw, Poland

## Abstract

Diabetes is a lifelong course disease, so insulin treatment has to be effective and safe, and patients should be satisfied with it. We aimed to compare efficacy, safety, and quality of treatment satisfaction of human and premixed analogue insulin among 3264 patients (53.58% women) with type 2 diabetes mellitus (T2DM) in a real-life environment. 2493 patients (62.77%) had been assigned to group I where before the inclusion into the study the treatment regimen has been changed from analogue to human premixed insulin and 771 patients (37.23%) to group II where the treatment with insulin analogue remained unchanged. At the end of the study, there was a reduction of HbA1c observed in both of the groups; however, Δ HbA1c was significantly higher in group 1 (−0.599 versus −0.406; *P* < 0.001 at visit 3 versus visit 1). The number of hypoglycemic episodes during the study observation was insignificantly reduced in both groups. Diabetes treatment satisfaction measured with DTSQ increased at the end of the study and was significantly better in group I compared to group II (*P* < 0.001). This observational study proved that both human and premixed analogue insulin are effective and safe, and patients are satisfied with the treatment.

## 1. Introduction

Insulin treatment is a necessity of life for many patients with T2DM [1]. After almost 100 years post insulin invention, it is still a cornerstone for type 2 diabetes treatment to maintain an optimal blood glucose level. Since the disease is not well controlled in many patients [2], as a response, new insulin analogues and different insulin treatment regimens were raised to cope with it [3]. On the other hand, it must be noted that new insulin formulations generate higher costs, which may limit their use [4]. When the decision regarding choosing the right treatment agent is made, there are always the same issues that have to be addressed namely, that the treatment must not only be effective, but also safe and patients have to be satisfied with it. In order to fulfill those prerequisites and due to the lifelong course of diabetes, the insulin therapy must be chosen individually in relation to patient's needs, treatment goals, and safety [5].

Treatment of patients who use basal insulin and require additional mealtime insulin can be intensified by an addition of a mealtime short-acting human insulin or by switching to a premixed insulin regimen [5]. The treatment option first mentioned can be in the form of basal-plus regimen, but usually full basal-bolus is required with time, which can be challenging for patients [5, 6]. The latter formulations combine a fixed combination of short-acting regular insulin or rapid-acting insulin analogue with an intermediate-acting protamine component providing mealtime and basal blood glucose control in one injection, which offers a more convenient however less flexible form of treatment in terms of titration and timing and requires a regulated lifestyle [1, 7]. Premixed insulin formulations are among the most frequently used in many countries [8]. There are apparent differences in pharmacokinetic and pharmacodynamics properties between premixed insulin analogues and conventional premixed human insulin [9]. Whether the differences possess a clinical importance remains a matter of discussion and surely depends on an individual patient clinical condition [10]. However, insulin analogues may not be suitable for all patients with diabetes. For many patients, a disadvantage regarding the application of insulin analogue may be a therapy expense [10, 11] as well as too short time of action among individuals by whom insulin formulation requires a longer time span, for example, those who used to eat snacks. It is very important to implement insulin therapy to patients who are likely to adhere, because nonadherence to pharmacotherapy has been linked to unfavorable outcomes [12]. Not one of the observational studies regarding premixed insulin therapy performed to date aimed at comparing switching premixed insulin analogue therapy to human one even though it might be a real-life therapeutic option in the light of guidelines of therapy individualization [8, 13].

To our best knowledge, there have been no studies performed up to date which aimed to observe an everyday life diabetologists' decision made regarding insulin treatment with a special focus on efficacy, safety, and patient quality of treatment satisfaction when premixed insulin analogues were switched to premixed human insulin in comparison to continuation of treatment with premixed insulin analogue.

## 2. The Aim of the Study

This observational study, PROGENS BENEFIT, aimed to compare efficacy, safety, and quality of treatment satisfaction of human and premixed analogue insulin among type 2 diabetic patients.

## 3. Materials and Methods

The PROGENS BENEFIT was a 24-week prospective, observational, multicenter study performed among 3264 adult T2DM patients who had been treated with metformin and premixed human or analogue insulin. Patients were recruited from 166 outpatient diabetology clinics distributed all over Poland. Each of the 109 diabetologists participating in the study recruited approximately 30 consecutive patients seen on a regular visit fulfilling the inclusion/exclusion criteria. Due to observational character of the study, there were no defined study procedures and all assessments were performed by the diabetologists as a part of routine clinical care. At first visit, there were demographic data recorded: age, gender, locality of living, school education level, time of diabetes duration, type of insulin treatment, and number and severity of hypoglycemic episodes occurring 4 weeks prior to the baseline visit. There were also measurements taken: body mass and height in kilograms and meters, respectively, and body mass index (BMI) calculated as a weight in kilograms divided by height in square meters. Data on HbA1c, postprandial plasma glucose (PPG) and fasting plasma glucose (FPG) were obtained from the results of tests performed as part of the patients' routine management of diabetes. FPG and PPPG values obtained within the last week and HbA1c values obtained within the last 4 weeks prior to each visit were considered acceptable.

The inclusion criteria were as follows: patients with type 2 diabetes diagnosed at least one year prior inclusion to the study based on clinical criteria [13], 18 years of age or above, treated with metformin in a stable dose of ≥1500 mg throughout a minimum of one month and premixed human 30/70 or 50/50 insulin (Gensulin M30, Gensulin M50) or analogue 30/70 or 25/75 or 50/50 insulin with daily insulin recruitment exceeding 30 IU and a stable dose for a minimum of one month before the inclusion into the study, HbA1c > 7%, and BMI <40 kg/m^2^.

The exclusion criteria included diabetes other than type 2, a history or presence of serious cardiovascular disease (myocardial infarction/acute coronary event or stroke in the last 3 months, heart failure in NYHA stage IV and coronary heart disease according to CCS in grade 3 or 4), unstable hypertension (>180/100 mm Hg) despite antihypertensive drug use, impaired renal function (eGFR < 30 ml/min, creatinine concentration > 135 *μ*mol/l), severe hepatic dysfunction (the aspartate transaminase and alanine transaminase activities exceeding the normal range 3 times above the upper limit of the normal range), medication with systemic glucocorticosteroids (excluding inhaled preparations), ACTH or interferon, chronic mental disorders, addiction to alcohol or drugs, participation in other clinical trials during the preceding 3 months, allergy to insulin or any other compounds of the preparation, pregnancy or breastfeeding, and other conditions or diseases that could be considered a contraindication for participating in the trial and human insulin therapy (Gensulin M30 or Gensulin M50) for longer than 4 weeks.

After the first enrollment into the study visit, patients attended two follow-up visits, every 12 weeks ± 7 days, so that the patients were observed over a total of 24 weeks. After recruitment, patients have been divided into two groups. Group I consisted of patients who had been treated with insulin analogue 30/70 or 25/75or 50/50, and at the discretion of specialists in diabetology, the treatment has been changed to human premixed insulin 30/70 or 50/50 (Gensulin M30 or Gensulin M50) in the time range between 2 to 4 weeks before the inclusion into the study. An insulin prescribed was administered twice with the dosage adjusted individually as required, and information about the dose was recorded at baseline, after 12 weeks and at the final visit (after 24 weeks). Group II consisted of patients who had been treated with insulin analogue, and the treatment was not changed to human premixed insulin in the time range between 2 and 4 weeks before the inclusion into the study. Patients had the dose adjusted individually as required in order to improve the blood glucose control.

All participating patients had been educated regarding behavioral treatment of diabetes (diet, physical activity) according to a routine local practice. The body mass was recorded at each study visit. Laboratory assessment of HbA1c was subject to local standardization and was performed before each study visit. The primary objective of the study was to assess the effectiveness and safety of the premix human and analogue insulin as an addition to metformin. The secondary objective of the study was to assess patients' treatment satisfaction with the use of validated and universally used Diabetes Treatment Satisfaction Questionnaires (DTSQ) [14] which was implemented as baseline and at the final visit. Effectiveness of insulin treatment was evaluated based on the change in HbA1c, FPG and PPG assessed at the final visit. Safety was assessed based on the number and severity of reported hypoglycemic episodes. Hypoglycemic episodes were analyzed during each study visit. Safety assessments included the change in number and severity of hypoglycemic events in the last 12 weeks prior to baseline and final visit. Hypoglycemic episodes were classified as severe or documented symptomatic hypoglycemia and pseudohypoglycemia. The definitions of hypoglycemic episodes were based on the report from the American Diabetes Association Workgroup on Hypoglycemia [15]. Severe hypoglycemia was defined as an episode requiring the assistance of another person to raise the plasma glucose concentration resulting in a resolution of symptoms, with or without a measured low plasma glucose concentration. Documented symptomatic hypoglycemia was defined as symptoms consistent with hypoglycemia with a measured plasma glucose concentration < 70 mg/dl (3.9 mmol/l). Pseudohypoglycemia was defined as typical symptoms of hypoglycemia with a measured plasma glucose concentration > 70 mg/dl (3.9 mmol/l) but approaching the level of hypoglycemia.

The statistical analysis was performed using Statsoft Statistica v12 software. In the case of quantitative variables, normality of distribution was tested by Shapiro-Wilk test. Numerical data were described as average ± standard deviation and categorical outcomes were presented as percentages. Distribution of quantitative variables was evaluated based on the average and standard deviation, while the distribution of categorical variables was presented using percentage. Mann–Whitney *U* test and Student's *t*-test depending on the number of cases and normality distribution were used for statistical analysis of quantitative variables. Chi-square test and Fisher test were used for statistical analysis of categorical variables. Statistical significance level was set at *P* < 0.05.

The study was performed in accordance with the Declaration of Helsinki and was approved by the local Bioethics committee. All participants gave written informed consent to participate in the study. The manuscript was prepared according to STROBE guidelines [16].

## 4. Results

**Figure 1:**
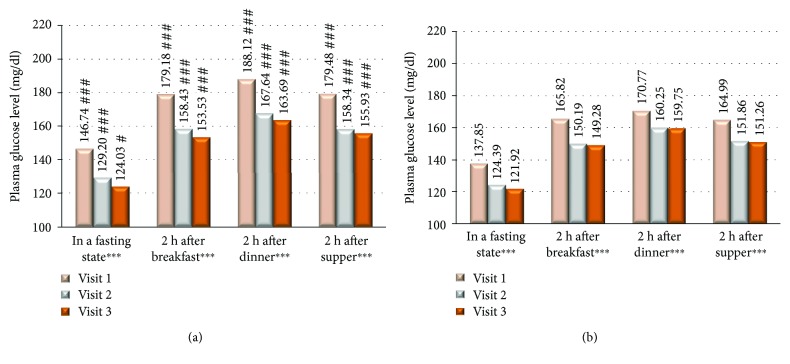
(a) Mean FPG and PPG among group I at each visit. (b) Mean FPG and PPG among group II at each visit. ^∗∗∗^*P* < 0.001 statistically significant difference between the 1st and 3rd visits. ^#^*P* < 0.05 and ^###^*P* < 0.001 statistically significant differences between group I and group II. h: hours; FPG: fasting plasma glucose (FPG); PPG: postprandial plasma glucose.

**Figure 2:**
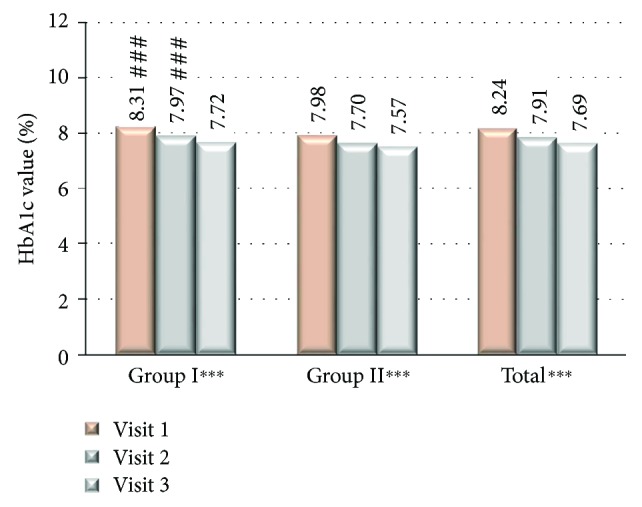
HbA1c value at each site visit. ^∗∗∗^*P* < 0.05 statistically significant difference between the 1st and 3rd visits. ^###^*P* < 0.05 statistically significant difference between group I and group II.

**Figure 3:**
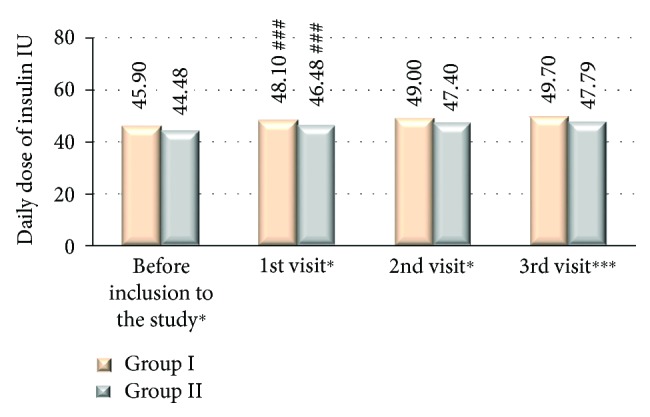
Daily dose of insulin at each visit. ^∗^*P* < 0.05, ^∗∗^*P* < 0.01, and ^∗∗∗^*P* < 0.001 statistically significant differences between groups I and II. ^###^*P* < 0.001 statistically significant difference between the 1st and 2nd visits.

**Figure 4:**
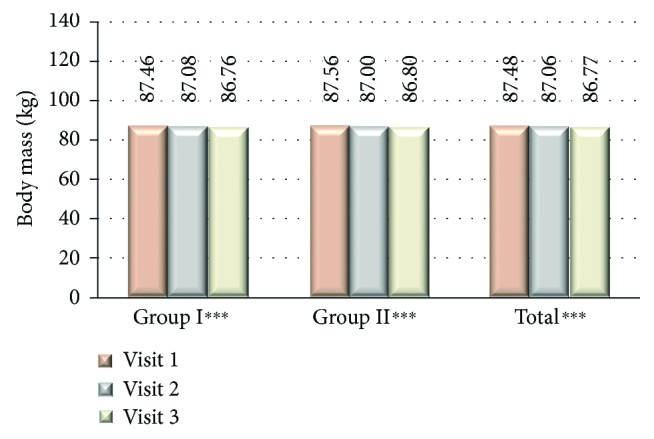
Body mass at each visit. ^∗∗∗^*P* < 0.05 statistically significant difference between the 1st and 3rd visits.

From the total of 3264 type 2 diabetic patients (53.58% women) with HbA1c > 7% and daily insulin dose > 30 IU, 2493 patients (76.37%) had been assigned to group I where the treatment regimen has been changed from analogue to human premixed insulin and 771 patients (23.62%) to group II where the treatment with insulin analogue remained unchanged. There were no significant differences in terms of patients' age, but there were significantly more women in the group I compared to the group II (Table 1). In the group I, compared to group II, there was a significantly higher number of patients from the rural area 29.61 versus 26.19%, *P* < 0.05, and less educated where basic education level was significantly lower in group I compared to group II (21.84 versus 16.69%, *P* < 0.01) and university level education was declared by significantly lower number of patients in group I compared to group II (5.72 versus 13.02%, resp., *P* < 0.001). There was a significant difference in FPG and PPG among studied groups both in between first and last visits as well as between group I and group II at each study visit where the mean glucose values were significantly higher in group I compared to group II (Figure 1). HbA1c values were reduced significantly in both studied groups; however, the difference was significant in group II compared to group I at visits 1 and 2 with no significant difference at last visit (Figure 2). At the end of the study, there was a reduction of HbA1c observed in both of the groups; however, ΔHbA1c was significantly higher in group I (−0.599 versus −0.406; *P* < 0.001 at visit 3 versus visit 1). Insulin dose differs significantly among the studied groups, and it was higher at all study visits in group I compared to group II as well as it differed significantly among groups comparing visit 1 and visit 2 (Figure 3). The metformin dose remained stable throughout the trial and did not differ significantly between the studied groups of patients. The number of hypoglycemic episodes during the study observation was insignificantly reduced in both groups. Cases of hypoglycemic episodes were less frequently observed in group II compared to group I (5.8 versus 6.0 episodes/per patient/per year (PPY), resp.). The opposite situation was in cases of severe hypoglycemia and pseudohypoglycemia, where both episodes were less frequently observed in group I than in group II (1.5 versus 1.9 episodes PPY, resp.) and as for pseudohypoglycemia (3.78 versus 3.8 episodes/per patient/per year, resp.). However, all of the differences were not statistically significant. There was a significant reduction in body mass weight observed between first and last visits in both studied groups (Figure 4). Diabetes treatment satisfaction measured with DTSQ increased at the end of the study and was significantly better in group I compared to group II (*P* < 0.001). During the study, there was a significant reduction in perception of hyperglycemic episode frequency in both groups (*P* < 0.001) and no change in perception of hypoglycemic episodes.

## 5. Discussion

The study performed in a large group of patients with type 2 diabetes mellitus proved that premixed insulin both analogue and human are efficient and safe, and studied patients were satisfied with the treatment method. It is important for clinicians to implement prescribing decisions and patient management based on well-designed studies according to evidence-based medicine preferably randomized controlled trials (RCT). Although the study is an observational one, it is currently becoming apparent that data from observational studies have become an increasingly important source of evidence because they reflect the local standards of medical care and have very simple inclusion/exclusion criteria although they observe a heterogeneous population reflecting everyday clinical setting [17, 18]. Because it was a noninterventional study, insulin therapies were prescribed by diabetologists according to a standard clinical practice and the data gave an insight into the model of diabetes care in real-life setting across Poland. We have focused on changing the treatment paradigm from insulin analogue to a human one because real-world data coming from everyday practice reveals that it is not an exception that patient using analogue insulin is switched to a human one. The potential reasons for the decision-making may be the longer time of human insulin action, which might be more suitable for patients eating snacks, and lower insulin cost, which is especially important for some groups of patients and as well as a country as a whole. It can only be a matter of speculation what the reasons were for the switch of insulin regimen in our study, but price may be an important one since majority of the patients treated with human insulin come from the rural areas and were less educated than patients treated with analogue insulin, so possibly they were people with lower incomes compared to the ones who are better educated and live in a city. While analyzing RCT, as well as observational studies, the studies performed to date aimed to discover the efficiency and safety of switching premixed insulin regimen from human to analogue one, but not vice versa [19–21]. Our real-word data proved that the two types of insulin regimens human and analogue were comparable in terms of hypoglycemia occurrence, and the frequency of any hypoglycemic episode was close to 6 episodes PPY, which is similar to the rate observed in other RWD studies ranging from 1.04 to 27 episodes PPY as summarized lately by Elliott et al. [22]. The difficulty in accurate determination of the frequency of hypoglycemic events is caused by differences in definitions of hypoglycemia used across studies and methods of collection of data on hypoglycemia occurrence. The dose of insulin was similar when compared to other real-world data (RWD) study, for example, PRESENT [23], but higher when compared to others like ASEAN [24] or IMPROVE [25]. In the presented study, the insulin dose was significantly higher among patients treated with human insulin compared to the ones using insulin analogue; however, it affected neither hypoglycemia rate nor BMI. In fact, body mass was reduced significantly in both studied groups which may be explained simply by participation in the study and willingness to obtain better results. Maintaining, however, not reducing BMI is in accordance with other observational study performed among Polish cohort of patients treated with biphasic human insulin [26]. Besides insulin therapy, patients were given support related to behavioral treatment of the disease, which is of extreme importance since it was proven in another large cohort of Polish patients with type 2 diabetes that their diet-related knowledge is insufficient and their physical activities are very low [27]. Both premixed insulin analogues and premixed human regimens were significantly effective in terms of HbA1c reduction; however, patients treated with insulin analogue obtained significantly better results in terms of reduction of both FPG, PPG, and HbA1c values. Higher reduction in FPG and PPG but not HbA1c observed in our study is in accordance with the other studies [28–30]. As proven in previously performed study by Roach et al., a reduction in HbA1c value was comparable among patients using premixed human or analogue insulin [31]. It is difficult to explain the nature of this phenomenon since patients remained on the same insulin regimen; however, it may be caused by intensification of current treatment and due to participation in the study by higher motivation to gain a better blood glucose control. Both studied groups were similarly satisfied with the insulin treatment measured with DTSQ. To our best knowledge, there are no studies that compare the treatment satisfaction among people switched from premixed analogue to premixed human insulin, but those performed on people switched from human to analogue indicate that the treatment modalities have a comparable treatment quality [19].

There are several limitations of the study that have to be addressed. First of all, the observational character of the study and lack of placebo group limit the evaluation of the effectiveness of the studied drugs because one cannot conclude that observed decrease of HbA1c was caused by insulin or simply by inclusion in the study. There was no information gathered regarding the reason why insulin was switched although possessing the information could bring a new light into an insulin treatment decision-making in a real-life clinical practice setting. Additionally, a diagnosis of hypoglycemia was solely dependent on the patients' ability to recall the event; hence, it could be regarded as biased. Despite limitations mentioned above, the data is clinically relevant because it provides data relating to a heterogeneous real-life local setting and proves that in an everyday practice it is not an exception that an analogue insulin is switched to a human one and that the changed regimen has proven to be effective and safe, and patients have been satisfied with it.

## 6. Conclusion

This observational study proved that changing treatment option from premixed analogue insulin to human one is a popular option in Poland, that insulin both analogue and human are efficient and safe, and that patients are satisfied with the treatment. Observational studies are important for clinicians because they are conducted in a real-world environment and provide valuable data regarding the use of a drug in a clinical practice not under the strict supervision of RCT. Because diabetes mellitus is a lifelong course disease, the choice of treatment method should take into account a number of aspects, among which are medication efficacy, safety, and patient satisfaction, and cost is of major importance.

## Figures and Tables

**Table 1:** Demographical and clinical characteristics of the studied groups.

Groups	Total *n* = 3264	Group I *n* = 2493	Group II *n* = 771	*P*
Male/female (%)	46.42/53.58	45.05/54.95	51.91/48.09	<0.002
Age (years) (±SD)	65.28 ± 9.29	65.47 ± 9.1	64.65 ± 8.9	NS
BMI (kg/m^2^) (±SD)	31.17 ± 4.16	31.31 ± 3.85	30.72 ± 4.01	NS
Baseline HbA1c (%; mmol/mol) (±SD)	8.24 ± 1.33	8.31 ± 1.28	7.98 ± 1.31	<0.001
	66.6 ± 10.7	67.3 ± 10.3	63.7 ± 10.6	
Diabetes duration (years) (±SD)	11.61 ± 6.28	11.58 ± 6.54	11.69 ± 6.72	NS
Insulin therapy duration (years) (±SD)	6.02 ± 4.55	5.97 ± 4.21	6.16 ± 4.31	NS

NS: statically insignificant; *n*: number of patients; SD: standard deviation; BMI: body mass index; HbA1c: glycated hemoglobin.

## References

[1] Turner R. C., Cull C. A., Frighi V., Holman R. R., UK Prospective Diabetes Study (UKPDS) Group (1999). Glycemic control with diet, sulfonylurea, metformin, or insulin in patients with type 2 diabetes mellitus: progressive requirement for multiple therapies (UKPDS 49). *JAMA*.

[2] Ford E. S. (2011). Trends in the control of risk factors for cardiovascular disease among adults with diagnosed diabetes: findings from the National Health and Nutrition Examination Survey 1999–2008^∗^. *Journal of Diabetes*.

[3] Danne T., Bolinder J. (2011). New insulins and insulin therapy. *International Journal of Clinical Practice*.

[4] Cameron C. G., Bennett H. A. (2009). Cost-effectiveness of insulin analogues for diabetes mellitus. *Canadian Medical Association Journal*.

[5] Inzucchi S. E., Bergenstal R. M., Buse J. B. (2012). Management of hyperglycaemia in type 2 diabetes: a patient-centered approach. Position statement of the American Diabetes Association (ADA) and the European Association for the Study of Diabetes (EASD). *Diabetologia*.

[6] Peyrot M., Barnett A. H., Meneghini L. F., Schumm-Draeger P. M. (2012). Insulin adherence behaviours and barriers in the multinational global attitudes of patients and physicians in insulin therapy study. *Diabetic Medicine*.

[7] Garber A. J., Ligthelm R., Christiansen J. S., Liebl A. (2007). Premixed insulin treatment for type 2 diabetes: analogue or human?. *Diabetes, Obesity & Metabolism*.

[8] Kalra S., Balhara Y. P., Sahay B. K., Ganapathy B., Das A. K. (2013). Why is premixed insulin the preferred insulin? Novel answers to a decade-old question. *The Journal of the Association of Physicians of India*.

[9] Mudaliar S. R., Lindberg F. A., Joyce M. (1999). Insulin aspart (B28 asp-insulin): a fast-acting analog of human insulin: absorption kinetics and action profile compared with regular human insulin in healthy nondiabetic subjects. *Diabetes Care*.

[10] Davidson M. B. (2014). Insulin analogs—is there a compelling case to use them? No!. *Diabetes Care*.

[11] Palmer J. L., Knudsen M. S., Aagren M., Thomsen T. L. (2010). Cost-effectiveness of switching to biphasic insulin aspart from human premix insulin in a US setting. *Journal of Medical Economics*.

[12] Currie C. J., Peyrot M., Morgan C. L. (2012). The impact of treatment noncompliance on mortality in people with type 2 diabetes. *Diabetes Care*.

[13] Marathe P. H., Gao H. X., Close K. L. (2017). American Diabetes Association standards of medical care in diabetes 2017. *Journal of Diabetes*.

[14] Bradley C., Lewis K. S. (1990). Measures of psychological well-being and treatment satisfaction developed from the responses of people with tablet-treated diabetes. *Diabetic Medicine*.

[15] Seaquist E. R., Anderson J., Childs B. (2013). Hypoglycemia and diabetes: a report of a workgroup of the American Diabetes Association and the Endocrine Society. *Diabetes Care*.

[16] Elm E. V., Altman D. G., Egger M. (2007). Strengthening the reporting of observational studies in epidemiology (STROBE) statement: guidelines for reporting observational studies. *BMJ*.

[17] Ligthelm R. J., Borzi V., Gumprecht J., Kawamori R., Wenying Y., Valensi P. (2007). Importance of observational studies in clinical practice. *Clinical Therapeutics*.

[18] Sahay B. K., Seshiah V. (2013). Importance of observational studies in understanding regional clinical practice: rationale and design of the A1chieve study. *The Journal of the Association of Physicians of India*.

[19] Yamada S., Watanabe M., Kitaoka A. (2007). Switching from premixed human insulin to premixed insulin lispro: a prospective study comparing the effects on glucose control and quality of life. *Internal Medicine*.

[20] Akhtar S., Shetty R., Kumar A., Das A. K., Kalra S. (2015). Clinical experience of switching from biphasic human insulin to biphasic insulin aspart 30 in Indian patients with type 2 diabetes in the A_1_chieve study. *Indian Journal of Endocrinology and Metabolism*.

[21] Hussein Z., Lim-Abrahan M. A., Jain A. B., Goh S. Y., Soewondo P. (2013). Switching from biphasic human insulin to biphasic insulin aspart 30 in type 2 diabetes: results from the ASEAN subgroup of the A_1_chieve study. *Diabetes Research and Clinical Practice*.

[22] Elliott L., Fidler C., Ditchfield A., Stissing T. (2016). Hypoglycemia event rates: a comparison between real-world data and randomized controlled trial populations in insulin-treated diabetes. *Diabetes Therapy*.

[23] Shestakova M., Sharma S. K., Almustafa M. (2007). Transferring type 2 diabetes patients with uncontrolled glycaemia from biphasic human insulin to biphasic insulin aspart 30: experiences from the PRESENT study. *Current Medical Research and Opinion*.

[24] Lim-Abrahan M. A., Jain A. B., Bebakar W. M. W., Seah D., Soewondo P. (2013). Safety and effectiveness of biphasic insulin aspart 30 in type 2 diabetes: results from the ASEAN cohort of the A_1_chieve study. *Diabetes Research and Clinical Practice*.

[25] Gumprecht J., Zurawska G., Wolnik B., Dzida G. (2008). The IMPROVE™ study - a multinational, observational study in type 2 diabetes: data from the polish cohort. *Endokrynologia Polska*.

[26] Walicka M., Jóźwiak J., Rzeszotarsk J. (2016). PROGENS-HbA_1c_ study: safety and effectiveness of premixed recombinant human insulin (Gensulin M30). *Archives of Medical Science*.

[27] Klupa T., Możdżan M., Kokoszka-Paszkot J. (2016). Diet-related knowledge and physical activity in a large cohort of insulin-treated type 2 diabetes patients: PROGENS ARENA study. *International Journal of Endocrinology*.

[28] Hermansen K., Colombo M., Storgaard H., Ostergaard A., Kolendorf K., Madsbad S. (2002). Improved postprandial glycemic control with biphasic insulin aspart relative to biphasic insulin lispro and biphasic human insulin in patients with type 2 diabetes. *Diabetes Care*.

[29] McSorley P. T., Bell P. M., Jacobsen L. V., Kristensen A., Lindholm A. (2002). Twice-daily biphasic insulin aspart 30 versus biphasic human insulin 30: a double-blind crossover study in adults with type 2 diabetes mellitus. *Clinical Therapeutics*.

[30] Mattoo V., Milicevic Z., Malone J. K. (2003). A comparison of insulin lispro Mix25™ and human insulin 30/70 in the treatment of type 2 diabetes during Ramadan. *Diabetes Research and Clinical Practice*.

[31] Roach P., Yue L., Arora V. (1999). Improved postprandial glycemic control during treatment with Humalog Mix25, a novel protamine-based insulin lispro formulation. Humalog Mix25 study group. *Diabetes Care*.

